# How *CYP2D6* Polymorphism Modulates the Community-Wide Risk of *Plasmodium vivax* Infection: A Panel Study in Amazonian Brazil

**DOI:** 10.1093/infdis/jiaf412

**Published:** 2025-08-19

**Authors:** Maria Carolina Silva de Barros Puça, Isabela Marques Naziazeno, Viviane Cristina Fernandes dos Santos, Priscila Thihara Rodrigues, Priscila Rodrigues Calil, Winni Alves Ladeia, José Pedro Gil, Marcelo Urbano Ferreira, Tais Nobrega De Sousa

**Affiliations:** Molecular Biology and Malaria Immunology Research Group, Instituto René Rachou, Fundação Oswaldo Cruz (FIOCRUZ), Belo Horizonte, Minas Gerais, Brazil; Department of Microbiology, Tumor and Cell Biology, Karolinska Institutet, Solna, Sweden; Molecular Biology and Malaria Immunology Research Group, Instituto René Rachou, Fundação Oswaldo Cruz (FIOCRUZ), Belo Horizonte, Minas Gerais, Brazil; Molecular Biology and Malaria Immunology Research Group, Instituto René Rachou, Fundação Oswaldo Cruz (FIOCRUZ), Belo Horizonte, Minas Gerais, Brazil; Institute of Biomedical Sciences, University of São Paulo, São Paulo, Brazil; Institute of Biomedical Sciences, University of São Paulo, São Paulo, Brazil; Institute of Biomedical Sciences, University of São Paulo, São Paulo, Brazil; Department of Microbiology, Tumor and Cell Biology, Karolinska Institutet, Solna, Sweden; Clinical Tropical Medicine, Global Health and Tropical Medicine (GHTM), Institute of Hygiene and Tropical Medicine, NOVA University of Lisbon, Lisbon, Portugal; Institute of Biomedical Sciences, University of São Paulo, São Paulo, Brazil; Global Health and Tropical Medicine (GHTM) Associate Laboratory in Translation and Innovation Towards Global Health (LA-REAL), Institute of Hygiene and Tropical Medicine, NOVA University of Lisbon, Lisbon, Portugal; Molecular Biology and Malaria Immunology Research Group, Instituto René Rachou, Fundação Oswaldo Cruz (FIOCRUZ), Belo Horizonte, Minas Gerais, Brazil; Department of Microbiology, Tumor and Cell Biology, Karolinska Institutet, Solna, Sweden

**Keywords:** malaria, *Plasmodium vivax*, CYP2D6, primaquine, risk of infection

## Abstract

**Background:**

The CYP2D6 enzyme plays a critical role in the metabolism of primaquine, the most widely used drug for the radical cure of *Plasmodium vivax* malaria. Impaired CYP2D6 activity has been associated with an increased risk of relapse. However, the overall impact of CYP2D6 on infection dynamics is still not fully understood. We hypothesized that individuals with impaired CYP2D6 activity develop partial immunity more rapidly due to the ineffective clearance of hypnozoites.

**Methods:**

To test this hypothesis, we conducted a community-based study involving ∼1300 individuals genotyped for *CYP2D6* and assessed repeatedly for *P. vivax* using molecular diagnosis. This approach allowed us to detect and monitor submicroscopic and asymptomatic infections over a 4-year follow-up period.

**Results:**

In our cohort, children with impaired CYP2D6 activity exhibited a higher frequency of *P. vivax* infections compared with those with normal enzyme activity. This pattern changed during the second decade of life, as the prevalence of *P. vivax* infection increased in adolescents with normal enzyme activity (*P* = .0008, Generalized additive mixed model). Consistent with this, parasite densities were lower in adults with impaired CYP2D6 activity compared with younger individuals with normal enzyme activity (*P* = .0383, Linear mixed model).

**Conclusions:**

These findings underscore the potential role of CYP2D6 in shaping infection dynamics and malaria immunity in endemic areas.

## Teaser

This study investigated whether individual variation in primaquine metabolism influences susceptibility to *P. vivax* by affecting hypnozoite clearance and immunity acquisition, revealing higher infection rates in children with impaired CYP2D6 activity and age-dependent shifts in infection patterns and parasite densities.

Malaria caused by *Plasmodium vivax* is the most prevalent form in the Americas, accounting for 72% of the 505 600 cases reported in 2023 [1]. In this context, Brazil contributes to ∼30% of the total number of cases in the Americas, with around 140 000 cases reported annually [2]. More than 99% of these cases occur in the Amazon region [3]. Although traditionally considered a predominantly rural disease, malaria is emerging in rapidly expanding urban centers within the Amazon region [4]. This trend was driven in part by intense rural-to-urban migration, with ∼72% of its population now living in settlements classified as urban [5].

*Plasmodium vivax* poses challenges for control and elimination, in part due to its ability to form dormant liver stages (hypnozoites) and transmit infective gametocytes to mosquitoes early in the infection. Beyond its biology, infection risk is highly heterogeneous and shaped by sociodemographic, genetic, and behavioral factors [6]. A small proportion of high-risk individuals contribute disproportionately to the malaria burden, even in low-transmission areas like the Amazon, where 20% of the population accounts for 86% of cases [7]. Repeated infections in these high-risk individuals lead to the gradual development of clinical immunity, contributing to an asymptomatic reservoir that sustains transmission despite low overall incidence [8].

The factors that affect the survival and activation of hypnozoites, in particular, can modulate the individual risk of infection and disease. These dormant liver-stage parasites can reactivate weeks or months after the initial infection, leading to relapses that contribute significantly to the disease recurrence and transmission [9]. In Brazil, *P. vivax* recurrences typically occur approximately two and a half months after treatment with chloroquine and primaquine, accounting for an estimated 20%–40% of all reported cases [10–12]. This highlights the critical importance of targeting hypnozoites to reduce disease burden and transmission [9].

Primaquine (PQ), an 8-aminoquinoline, is the most widely used drug for eliminating hypnozoites and preventing *P. vivax* recurrences [13, 14]. The World Health Organization (WHO) recommends a 14-day or 7-day PQ regimen combined with a blood schizontocide, such as chloroquine or artemisinin-based combination therapy [15]. Due to the risk of hemolysis, glucose-6-phosphate dehydrogenase (G6PD) testing is recommended before PQ administration. In line with WHO guidance, the Brazilian Ministry of Health has adopted the 7-day regimen to improve adherence without compromise efficacy [10]. Tafenoquine (TQ), another 8-aminoquinoline, has been introduced as an alternative to PQ for the radical cure of *P. vivax* malaria [16]. With a longer half-life, TQ enables single-dose administration, potentially improving adherence [17].

It is well documented that PQ requires activation by the cytochrome P450 2D6 (CYP2D6) enzyme to exert its effect [14, 18]. The gene that encodes the CYP2D6 enzyme is highly polymorphic, with over 160 known allelic variants that determine metabolic phenotypes, ranging from poor to ultrarapid metabolizers [19]. This broad spectrum of enzymatic activity influences the effectiveness of PQ, which varies according to each individual's phenotype [20, 21]. Decreased CYP2D6 activity has been associated with PQ treatment failure and increased relapse rates, particularly in the Brazilian Amazon region [11, 22]. Unlike PQ, the role of CYP2D6 in TQ metabolism remains unclear, with conflicting evidence from murine models [23, 24] and limited data in humans regarding its impact on antihypnozoite activity [17].

We investigated whether individual variation in PQ metabolism modulates susceptibility to *P. vivax*. Our hypothesis is that impaired CYP2D6 activity leads to suboptimal hypnozoite clearance and more frequent relapses, thereby accelerating the acquisition of partial immunity to *P. vivax*. To test this, we conducted a community-based study in the Brazilian Amazon, with 7 survey waves over 4 years. We genotyped *CYP2D6* and inferred enzyme activity in ∼1300 individuals, repeatedly tested using molecular diagnostics to detect and track submicroscopic and asymptomatic infections. To our knowledge, this is one of the largest studies to assess the impact of CYP2D6 metabolism on *P. vivax* infection risk.

## METHODS

### Study Site and Population

This study was conducted in Mâncio Lima, Acre, Brazil, a municipality located in the Alto Juruá region near the Peruvian border. As part of a longitudinal cohort initiated in 2018, ∼20% of the local population was enrolled through simple random sampling of households enumerated in a 2015–2016 baseline census [25]. Participants were followed in 7 semiannual surveys, conducted at 6-month intervals between April 2018 and November 2021, to assess malaria transmission dynamics in the urban area, where nearly half of the cases are locally acquired, and *Plasmodium vivax* predominates [25, 26]. Finger-prick blood samples and sociodemographic data were collected at each time point. The DNA was extracted using DNA Investigator kits (Qiagen, Hilden, Germany) and a QIASymphony automated platform (Qiagen). *Plasmodium vivax* infections diagnosed by microscopy were treated with chloroquine (total dose, 25 mg/kg over 3 days) and primaquine (0.5 mg/kg/day for 7 days) [8]. Consistent with current national guidelines in Brazil, G6PD deficiency screening was not routinely performed prior to primaquine treatment [27]. Detailed descriptions of the study site, participant selection, data collection, DNA extraction, and diagnosis are provided in the Supplementary Material.

### Ethical Approvals and Participant Consent

The ethical and methodological aspects of part of this study were approved by the Human Research Ethics Committee of the René Rachou Research Center (CAAE 91229218.6.0000.5091). The project is also approved by the Research Ethics Committee of the Institute of Biomedical Sciences at the University of São Paulo and the National Research Ethics Committee of the Brazilian Ministry of Health (CAAE numbers 64767416.6.0000.5467 and 30481820.3.0000.5467).

### *CYP2D6* Polymorphism Genotyping and Copy Number Analysis

*CYP2D6* genotyping was performed using the OpenArray platform (Applied Biosystems), following the manufacturer's recommendations. A panel of 12 CYP2D6 polymorphisms was selected based on their frequency and functional relevance in the Brazilian population [11, 28], following PharmVar and CPIC guidelines [19]. A detailed list of the selected variants and their functional impact is provided in the Supplementary Material. *CYP2D6* copy number variations (CNVs) were assessed by qPCR (Hs00010001_cn TaqMan assay) using RNase P as reference gene and estimated with CopyCaller® v.2.0. Hybrid alleles were not distinguished due to methodological limitations. Detailed protocols are provided in the Supplementary Material.

### Prediction of the *CYP2D6* Phenotype Based on the AS Model

We inferred *CYP2D6* haplotypes using the PHASE v.2.1, executing the algorithm with 50 000 burn-in steps, followed by 400 000 iterations and a thinning interval of 1000 iterations. Convergence was evaluated by performing duplicate runs and comparing outputs, which yielded identical haplotype assignments, confirming the consistency of the results. The inferred haplotypes were compared with those in the PharmVar database for allele designation (https://www.pharmvar.org/gene/CYP2D6).

Each allele was assigned a metabolic activity score relative (AS) to CYP2D6*1, based on PharmVar and CPIC guidelines [19]: 1 for fully functional, 0 for nonfunctional, and 0.25–0.5 for reduced-function alleles. Scores were multiplied for duplicated/multiplied alleles and summed to derive genotype-predicted phenotypes: poor (gPM, AS = 0), intermediate (gIM, AS = 0.25–1), normal (gNM, AS = 1.25–2.25), and ultrarapid metabolizers (gUM, AS > 2.25). More details are provided in the Supplementary Material.

### Duffy Antigen/Receptor for Chemokines Genotyping

We considered the Duffy antigen/receptor for chemokines (DARC) genotype in the infection risk analysis, as *P. vivax* relies on this receptor to invade reticulocytes. Individuals with the Duffy-negative phenotype are primarily characterized as resistant to *P. vivax* infections [29]. Genotyping was performed according to a previously published protocol [30]. TaqMan assays were used to genotype two DARC polymorphisms: the T-33C substitution in the RBC-specific GATA1 transcription factor binding motif (rs2814778), which suppresses DARC expression on the RBC surface (FY*B^ES^ allele), and the G125A polymorphism (rs12075), which distinguishes the FY*B (wild-type) and FY*A (mutated) alleles.

### Statistical Analysis

The primary outcome was a *P. vivax* infection, defined primarily by species-specific qPCR targeting mitochondrial DNA or by microscopy in cases where qPCR was negative, regardless of the presence of symptoms. The second outcome was parasite density (in copies/µL) estimated by qPCR. This study aimed to evaluate whether impaired CYP2D6 was associated with the risk of infection and parasite densities in *P. vivax* malaria.

We estimated the probability of *P. vivax* infection as a function of CYP2D6 status by fitting generalized additive mixed models (GAMM). Unadjusted odds ratios (ORs) with 95% CIs were estimated by GAMM without covariates (“model 0”). CYP2D6 activity was included as a dichotomous variable: AS ≤ 1.0 (gPM/gIM) or AS > 1.0 (gNM/gUM). A smooth spline term was used to model age-specific effects. The final model included predictors previously associated with *P. vivax* infection risk in this population, such as wealth index (in quartiles) and study wave (7 categories) [8, 31]. Covariates were selected for inclusion in the regression models if associated with the outcome at the 15% significance level in exploratory unadjusted analysis. Neighborhood and individual identification were incorporated into the model as random effects to account for data clustering (residence) and repeated measures (participation in different cohorts). GAMM analysis was performed using the *gam* function in the mgcv package (RStudio v.2024.04.2). The goodness of fit was assessed by comparing the deviance of the candidate models and by Akaike information criterion (AIC) statistics.

We applied linear mixed models (LMM) to evaluate the relationship between CYP2D6 status and *P. vivax* parasite densities measured as the log of the number of amplicon copies per microliter, adjusting for potential confounders. The final model included age (4 categories), wealth index and study wave as predictors. The best-fitted model included an interaction term between age and CYP2D6 status. The variable “individual” was included as a random effect to account for repeated measures (participation in different cohorts). LMMs were fitted using the *lmer* function in the lme4 package (RStudio). The goodness of fit was assessed by comparing the deviance of the candidate models and by AIC statistics. We evaluated the model assumptions by testing for uniformity, dispersion (as a proxy for heteroskedasticity), and outliers using the *testResiduals* function in DHARMa within RStudio.

## RESULTS

### Frequency of CYP2D6 Variants in the Study Population

The data analyzed were part of the Mâncio Lima cohort conducted between 2018 and 2021, a larger study aimed to investigate epidemiological and clinical aspects of urban malaria in the Brazilian Amazon. The number of participants varied from 1394 in wave 1 to 2043 in wave 7 [8, 25]. We initially assayed 1862 individuals for 12 polymorphic sites in the *CYP2D6* gene by TaqMan OpenArray qPCR. The genotypes and predicted phenotypes were successfully determined for 1305 (70%) individuals. Many samples failed to amplify due to suboptimal DNA quality and/or insufficient concentration (Supplementary Figure 1).

We identified 14 alleles previously described by the PharmVar Consortium (Supplementary Table 1). The following alleles associated with decreased or no enzymatic activity were detected: *3, *4, *5, *6, *9, *10, *17, *29, and *41. Based on genotype data, the inferred metabolic phenotypes were: 4.1% (95% CI, 2.9–5.1) poor metabolizers (gPM), 23.5% (95% CI, 21.2–25.8) intermediate metabolizers (gIM), 61% (95% CI, 58.3–63.6) normal metabolizers (gNM), and 9% (95% CI, 7.4–10.5) ultrarapid metabolizers (gUM) (Supplementary Table 2). In a few cases, we could not predict the CYP2D6 phenotype; however, it was possible to determine the activity score and infer enzyme activity. In these cases, both alleles and the CNV were known. Thus, we identified 361 (27.7%) subjects carrying impaired enzyme activity (AS ≤ 1) and 944 (72.3%) showing normal (AS > 1) enzyme activity.

### CYP2D6 Status as a Risk Factor for Malaria Infection

The study population consisted of children and adults, with a median age of 24 years (IQR, 13–40), and was evenly distributed between genders (Table 1). During the 4-year cohort period, 946 (72.5%) subjects were screened for malaria parasitemia at least in 4 study waves, contributing to a total of 6309 samples. Using qPCR targeting mitochondrial DNA, the highest prevalences of *P. vivax* infection were detected at the beginning of the study in 2018 (7.6%–7.8%) and 2019 (6.3%) (Supplementary Table 3). The numbers decreased to 1.4% in the last wave in October–November 2021. *Plasmodium falciparum* malaria exhibited a similar trend, but with significantly fewer cases.

**Table 1.** Demographic and Genetic Characteristics of the Study Population and Malaria Prevalence in the Brazilian Amazon Region of Mâncio Lima, Acre State, 2018–2021

Characteristics	Study Population		
Age, y (median, IQR)	24 (13–40)		
Gender, *n* (%)			
Male	623 (47)		
Female	682 (52)		
Diagnoses all waves, *n* (%)			
Microscopy	*P. vivax*	*P. falciparum*	
Positive	23 (0.4)	6 (0.1)	
Negative	6296 (99.6)	6313 (99.9)	
qPCR	*P. vivax*	*P. falciparum*	
Positive	266 (4.4)	84 (1.3)	
Negative	6043 (95.6)	6225 (98.7)	
Diagnoses by age, *n*/*N*^a^	Impaired CYP2D6	Normal CYP2D6	OR (95% CI)^b^
0–14	8/397	30/1104	0.736 (0.289, 1.662)
15–34	19/485	109/1477	0.512 (0.293, 0.849)*
35–54	16/423	41/958	0.879 (0.455, 1.623)
>55	8/230	16/493	1.074 (0.392, 2.708)

Abbreviations: CI, confidence interval; OR, odds ratio.

^a^ *n*, number of qPCR-positives; *N*, total number of qPCR tests performed.

^b^Unadjusted OR.

^*^ *P* = .0077.

Most infections detected by qPCR were submicroscopic, with only 23 and 7 cases of *P. vivax* and *P. falciparum*, respectively, being microscopically detected over the 7 waves (Table 1). Additionally, most qPCR-positive infections were asymptomatic infections (Supplementary Table 3). The proportion of asymptomatic qPCR-positive infections varied from 87.8% in wave 1 to 92.9% in wave 7. Only 1 of the 66 DARC-negative (FY*B^ES^/FY*B^ES^) individuals was infected with *P. vivax*. As they are naturally resistant [29], these individuals were excluded from the risk factor analysis.

**Figure 1.**
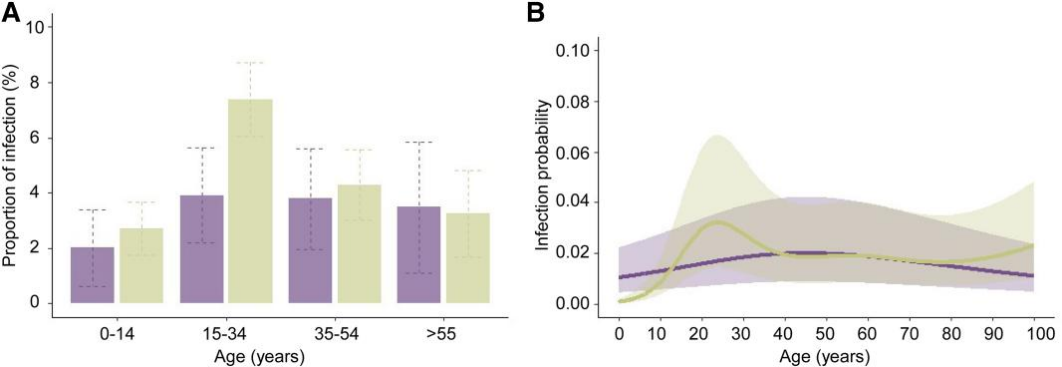
Impaired and normal CYP2D6 activity are represented in purple and green, respectively. (*A*) Prevalence of *Plasmodium vivax* malaria among different age groups and CYP2D6 status over a 4-y cohort period in Mâncio Lima, Acre, Brazil. Dashed lines represent 95% CI. (*B*) Probability of infection by *P. vivax* according to age and inferred CYP2D6 phenotype status in the Mâncio Lima cohort. A GAMM was fitted to predict the probability of infection (qPCR positive, regardless of symptoms), adjusted for confounding factors. The line in the graph represents the median, and the shaded areas indicate the interquartile range.

We next evaluated whether the CYP2D6 inferred phenotypic status was associated with the risk of *P. vivax* infection (qPCR-positive regardless of symptoms). There was a significant difference in the probability of infection in the 15- to 34-year age group (Table 1). A lower proportion of infection was detected in individuals with impaired CYP2D6 activity (3.92%; 95% CI, 2.19–5.64) compared with normal enzyme activity (7.38%; 95% CI, 6.05–8.71) (OR = 0.512, 95% CI, 0.293–0.849, *P* = .0077) (Figure 1*A* and Table 1).

This association was further examined using a GAMM. To account for nonlinear relationships, we included a smooth function of age for each CYP2D6 category. Since most participants were from the Amazon region, age was used as a proxy for cumulative malaria exposure. Additionally, factors previously identified as associated with *P. vivax* infection in this population [8], such as wealth status and survey period, were incorporated into the analysis. An interesting finding was the low probability of infection in children with normal enzyme activity. However, this probability increases from adolescence to young adulthood before declining in the fourth decade of life (*P* = .0008) (Figure 1*B* and Table 2). In contrast, no significant changes were observed in the probability of infection among individuals with impaired CYP2D6 enzyme (*P* = .5133). Additionally, the prevalence of *P. vivax* decreased from waves 4–7, showing a lower chance of infection among wealthier residents (Table 2).

**Table 2.** Factors Associated With *Plasmodium vivax* Infection in Mâncio Lima Cohort, From 2018 to 2021

Variable	Model 0^a^			Model 1^b^		
	Odds Ratio	95% CI	*P*-value	Odds Ratio	95% CI	*P*-value
Study wave number and dates						
1. Apr–May 2018	Reference	…		Reference	…	
2. Sep–Oct 2018	0.903	0.554, 1.471	0.6822	0.930	0.568, 1.520	0.7721
3. May–June 2019	0.694	0.419, 1.147	0.1541	0.683	0.411, 1.134	0.1400
4. Sep–Oct 2019	0.343	0.198, 0.595	0.0001	0.348	0.200, 0.605	0.0002
5. Oct–Nov 2020	0.309	0.174, 0.546	<0.0001	0.297	0.167, 0.528	<0.0001
6. Apr–May 2021	0.133	0.067, 0.265	<0.0001	0.128	0.064, 0.255	<0.0001
7. Oct–Nov 2021	0.122	0.062, 0.243	<0.0001	0.113	0.057, 0.226	<0.0001
Wealth index quartile						
1. Poorest	Reference	…		Reference	…	
2.	0.698	0.458, 1.065	0.0958	0.586	0.369, 0.929	0.0229
3.	0.484	0.293, 0.800	0.0047	0.423	0.248, 0.722	0.0016
4. wealthiest	0.408	0.224, 0.740	0.0032	0.299	0.157, 0.570	0.0002
CYP2D6 status						
Normal	Reference	…		…	…	
Impaired	0.742	0.480, 1.146	0.1780	…	…	
Age group (y)						
0–14	Reference	…		…	…	
15–34	2.393	1.509, 3.796	0.0002	…	…	
35–54	1.634	0.962, 2.777	0.0695	…	…	
>55	1.201	0.604, 2.388	0.6021	…	…	

Abbreviation: CI, confidence interval.

^a^Results for unadjusted analysis are presented under “model 0.” Number of observations: 5567.

^b^By generalized additive model with mixed effects at both individual and household levels, adjusted for survey period and wealth status. The model includes a smooth spline term for age for each category of CYP2D6 (*P* = .0008 for normal CYP2D6 and *P* = .5133 for impaired CYP2D6). Infection is defined as a positive genus-specific PCR result confirmed by species-specific quantitative PCR probe-based assay, regardless of any symptoms. A total of 5567 from 1156 participants of 574 households were included in the model after excluding DARC-negative and participants with missing data.

We also evaluated the factors associated with *P. falciparum* infection, as its treatment outcome is not influenced by CYP2D6 metabolism. As expected, the probability of infection remained unchanged with age across CYP2D6 groups (Supplementary Table 4).

### Variation in Parasite Density Associated With CYP2D6 Status

**Figure 2.**
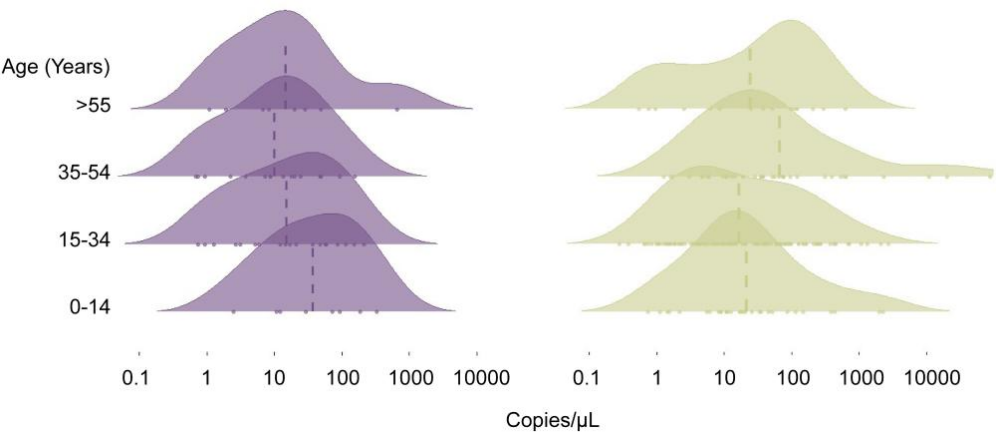
Parasite density distribution by age groups and CYP2D6 status in Mâncio Lima cohort. Vertical lines indicate geometric means. The dot represents an observation. Impaired and normal CYP2D6 activity are represented in purple (left panel) and green (right panel), respectively.

We further examined parasite densities, as measured by qPCR, in relation to CYP2D6 status. Most *P. vivax* infections were characterized by the detection of few copies of the parasite target (geometric mean = 21.53 copies/µL; 95% CI, 16.23–28.57). We found a tendency for higher parasite densities in individuals with normal enzyme activity later in life, whereas those with impaired enzyme activity exhibited higher parasitemia earlier in life (Figure 2 and Supplementary Table 5).

We evaluated this association by a LMM, adjusting for confounding factors such as survey period and wealth status. The analysis revealed a significant relationship between parasite densities and CYP2D6, which is modified by age. Notably, individuals in late adulthood (35–54 years) with impaired CYP2D6 activity showed lower levels of parasitemia compared with younger subjects (0–14 years) with normal enzyme activity (*P* = .0383, Supplementary Table 6). That is, for individuals in late adulthood with low activity, the log of the parasitemia decreases by 0.482 units compared with the reference category.

## DISCUSSION

*Plasmodium vivax* malaria has imposed significant challenges to eliminate, in part due to its capacity to form hypnozoites and remain silent in the liver. Also, as transmission drops, the parasite becomes more undetectable to the standard microscopic diagnosis [8]. In this large population-based study, we present a new finding concerning to what extent the ability to metabolize PQ and respond to treatment may influence the likelihood of future infections.

The relationship between CYP2D6 status and the risk of relapses has been well established. Several studies have demonstrated a clear association between impaired CYP2D6 function and an increased risk of therapeutic failure due to *P. vivax* relapses, especially in Brazil, where PQ is recommended by treatment guidelines to achieve the radical cure [11, 21, 22, 32]. Notably, nonimmune individuals who do not respond adequately to treatment are at higher risk of relapse [11]. In our current study, we demonstrate a broader effect of CYP2D6-dependent metabolism of PQ on infection dynamics, particularly in a context where most infections were submicroscopic and asymptomatic.

One of the strengths of this study is the large number of individuals enrolled in each survey, with over 70% being repeatedly screened for malaria in a 4-year follow-up. We found a prevalence of 28% (95% CI, 25.2–30.1) for impaired CYP2D6 activity. This rate was consistent with previous studies conducted in the Brazilian Amazon, which reported frequencies ranging from 19% to 35% [11, 22, 28, 33]. In our cohort, children with impaired CYP2D6 activity were found to be more often infected by *P. vivax* than those with normal enzyme activity. This pattern changed during the second decade of life as the prevalence of *P. vivax* infection increased in adolescents and early adulthood with normal enzyme activity. Consistent with this, parasite densities followed a similar tendency with higher parasitemia in adults with normal CYP2D6 activity. Importantly, this association with *P. vivax* infection was most evident among individuals aged 15–34 years, suggesting that these effects may be age-dependent and should not be generalized across all age groups. We propose that children with impaired CYP2D6 activity may develop partial immunity more rapidly due to experiencing a greater number of recurrent infections during their childhood. As expected, we did not observe any association between CYP2D6 status and *P. falciparum* infections.

Although no immunological markers were directly assessed in this study, previous studies have shown that greater exposure to malaria transmission is associated with stronger naturally acquired antibody responses, which are in turn linked to a lower risk of clinical *P. vivax* malaria in the Amazon [11, 30] These findings support the hypothesis that repeated exposure, whether due to relapses or reinfection, may accelerate the development of clinical immunity.

Consistent with this, we observed a high prevalence of asymptomatic *P. vivax* infections in our cohort, likely reflecting the historically intense transmission in the region where the study was conducted. The Juruá Valley, where the municipality of Mâncio Lima is located, has emerged as a primary malaria transmission hotspot in the Brazilian Amazon in recent years, reporting one of the highest parasitic annual indices in the region [26]. Sustained exposure in such settings promotes the gradual development of clinical immunity, which reduces symptoms and often results in low-density and submicroscopic infections [8].

In highly endemic areas, the acquisition of immunity follows an age-dependent pattern, with younger individuals rapidly developing clinical immunity after repeated malaria episodes [34–38]. However, malaria transmission dynamics in the Brazilian Amazon are highly complex, reflecting the different endemic settings. Thus, while some studies indicate that both infection risk and disease severity decrease with age [28, 39], others show that children and adults have similar probabilities of remaining asymptomatic [40]. Interestingly, clinical immunity can develop even under low endemicity conditions in settlement communities, highlighting the adaptability of immune responses to different transmission intensities [41].

Chronic exposure to *P. vivax* has been associated with an increased risk of anemia [35] and malaria during pregnancy [42]. Additionally, asymptomatic carriers of *P. vivax* harbor mature gametocytes and can experimentally infect local malaria vectors [43, 44]. Estimates suggest that these carriers represent a significant reservoir of parasites, accounting for 28%–79% of mosquito infections [45]. This silent contribution to transmission has important implications for malaria elimination efforts, as these carriers sustain parasite circulation within communities, even in the absence of evident clinical symptoms.

This study has some limitations. We could not access the participants' past malaria clinical history, which could have provided valuable insights into previous malaria events and their impact on immunity. We were also unable to evaluate the risk of recurrences, as malaria incidence was not continuously monitored throughout the study. Furthermore, the low prevalence of qPCR-positive infections limited our understanding of acquired immunity and detects robust associations between CYP2D6 activity and infection outcomes, particularly in age groups with few positive cases. Additionally, as *P. vivax* accumulates largely in the spleen [46], peripheral parasite densities may underestimate total parasite burden, limiting their association with immune status.

**Figure 3.**
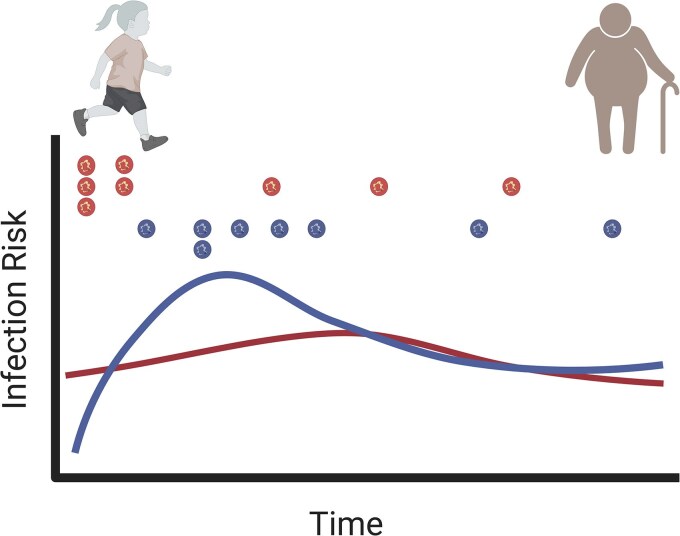
A schematic representation of the probability of *Plasmodium vivax* infection within the study population. Individuals with impaired CYP2D6 activity (curve in red) have a reduced ability to metabolize PQ, which leads to a higher risk of relapse (indicated by the stacked symbols). Over time, as immunity is gradually acquired, individuals with normal CYP2D6 activity become the most at-risk group to be infected (curve in blue) within the community. Symbols represent an infection. Created with BioRender.com. https://BioRender.com/smrbtt0.

In conclusion, we provide novel insights into the indirect effect of CYP2D6 metabolism on *P. vivax* infection risk, highlighting its role in treatment efficacy and malaria transmission dynamics. Our findings suggest that children with impaired CYP2D6 activity are at a higher risk of infection. Over time, while they gradually acquire immunity against infection, individuals with normal CYP2D6 activity become the most at-risk group from a community-level perspective (Figure 3). In other words, the speed at which an individual develops immunity may be influenced by the effectiveness of treatment, which could, in turn, affect their potential contribution to the parasite reservoir within the population. Thus, under specific conditions, individuals with normal CYP2D6 activity might contribute disproportionately to the reservoir of *P. vivax* and help sustain transmission. Further studies are required to confirm this hypothesis. These findings highlight the importance of integrating pharmacogenetics into malaria control strategies. Targeted interventions, such as molecular screening for submicroscopic infections, could be refined to account for variations in treatment response and associated epidemiological risk factors, ultimately improving malaria elimination efforts.

## Supplementary Material

jiaf412_Supplementary_Data

## Supplementary Data

Supplementary materials are available at *The Journal of Infectious Diseases* online (http://jid.oxfordjournals.org/). Supplementary materials consist of data provided by the author that are published to benefit the reader. The posted materials are not copyedited. The contents of all supplementary data are the sole responsibility of the authors. Questions or messages regarding errors should be addressed to the author.
