# Supplementary material for: How CYP2D6 Polymorphism Modulates the Community-Wide Risk of Plasmodium vivax Infection: A Panel Study in Amazonian Brazil
Source: J Infect Dis. 2025 Aug 19;232(4):e571–9. doi: 10.1093/infdis/jiaf412 (PMC12526952; doi:10.1093/infdis/jiaf412)
Supplement: jiaf412_Supplementary_Data [file jiaf412_supplementary_data.pdf]

SUPPLEMENTARY MATERIAL

Supplement to: **How *CYP2D6* polymorphism modulates the community-wide risk of *Plasmodium vivax* infection: a panel study in Amazonian Brazil**

Maria Carolina Silva De Barros Puça, Isabela Marques Naziazeno, Viviane Cristina Fernandes Dos Santos, Priscila Thihara Rodrigues, Priscila Rodrigues Calil, Winni Alves Ladeia, José Pedro Gil, Marcelo Urbano Ferreira, Tais Nobrega De Sousa

METHODS

Study site and population

The present study evaluated samples collected from individuals residing in Mâncio Lima, as part of a cohort study conducted in the municipality. Mâncio Lima (07°36'51"S, 72°53'45"W), is located in the Alto Vale do Juruá region, in the state of Acre, in the westernmost part of Brazil, near the border with Peru. At the start of the study in 2018, 48.7% of malaria cases in Mâncio Lima were reported as acquired in the urban area [1]. Eight public laboratories operating in healthcare units in the urban area provide free malaria diagnosis through microscopy. *Plasmodium vivax* accounted for 84.2% of locally acquired and laboratory-confirmed infections, 14.4% were caused by *P. falciparum,* and 1.4% by mixed-species infections [1,2].

The study was embedded in a population-based longitudinal cohort designed to monitor residual malaria transmission in urban areas. Detailed descriptions of the study area and design have been published elsewhere [3]. Briefly, a comprehensive population census was conducted between November 2015 and April 2016 to enumerate all households and residents. From this census, approximately 20% of the urban population was selected through simple random sampling to participate in the cohort [3–5], ensuring an unbiased representation, and a total of 2690 individuals were enrolled since April 2018. Baseline data collection occurred in April-May 2018 (wave 1), followed by subsequent surveys in September-October 2018 (wave 2), May-June 2019 (wave 3), September-October 2019 (wave 4), October-November 2020 (wave 5), April-May 2021 (wave 6), and October-November 2021 (wave 7), the original study design comprised semiannual waves, but that planned for April-May 2020 did not occur because of the COVID-19 pandemic [1,3]. Sociodemographic and morbidity information was collected and updated at each cohort of the study. Data on household assets were combined to derive a household wealth index [6]. Capillary blood samples were collected by finger prick from participants over three months of age for malaria diagnosis and genetic studies, regardless of the presence of symptoms [3]. To characterize clinical malaria, participants were first asked if they had experienced any signs or symptoms that could have been caused by a malaria infection. Those reporting signs or symptoms on the day of the interview or up to seven days prior were specifically asked about fever, chills, or headache, the triad of main clinical manifestations of uncomplicated malaria [7]. Additionally, during the COVID-19 pandemic period, individuals were also tested for SARS-CoV-2 to avoid any bias in infection diagnoses, as some symptoms, such as fever and headache, could be similar to those of malaria.

DNA extraction

DNA was extracted from 50 µL of whole blood using DNA Investigator kits (Qiagen, Hilden, Germany) and a QIASymphony automated platform (Qiagen). The final elution volume of DNA was 100 µL. Extractions were performed at the Malaria Immunity, Genomics, and Populations Laboratory (MOA) of São Paulo State University and stored at -20°C.

Malaria diagnosis and treatment

Samples were initially screened for malaria using a genus-specific qPCR assay performed with SYBR Green detection targeting a conserved region of the *cytb* gene of human-infecting *Plasmodium* species. Positive samples were subsequently evaluated using species-specific quantitative TaqMan assays (ThermoFisher Scientific). These assays targeted mitochondrial genome sequences of *P. vivax* (84-bp region of the *cox1* gene) and *P. falciparum* (90-bp region spanning the 3′ end of the *cox1* gene and the adjacent intergenic region). All qPCR amplification for malaria diagnosis was performed and described by Rodrigues and colleagues [1].

Plasmodium vivax infections identified were treated with chloroquine at a total dose of 25 mg/kg over three days, combined with primaquine at 3.5 mg/kg administered over seven days. In cases of *P. falciparum*, treatment consisted of a three-day regimen of artemether (2–4 mg/kg/day) and lumefantrine (12–24 mg/kg/day), along with a single 0.75 mg/kg dose of primaquine to eliminate gametocytes.

***CYP2D6* polymorphism genotyping and copy number analysis**

In order to phenotypically characterize the enrolled malaria patients, polymorphisms in the *CYP2D6* gene, selected from the literature (based on their frequency in the Brazilian population) by Silvino et al. and Ladeia-Andrade et al [8,9] and according to recommendations from the pharmacogenetics consortium [10], were chosen to be genotyped using the OpenArray platform (Applied Biosystems) with the Real-Time PCR method, following the manufacture recommendation. Using this platform, we were able to genotype 12 targets for 144 individuals in a single experiment.

The selected SNPs included nine polymorphic positions in the *CYP2D6* gene: G-1584C [rs1080985], G31A [rs769258], C100T [rs1065852], C1023T [rs28371706], G1846A [rs3892097], G2850 [rs16947], G2988A [rs28371725], G3183A [rs59421388], and G4180C [rs1135840]; as well as three deletions: 1707delT [rs5030655], 2549delA [rs35742686], and 2613_2615delAGA[rs5030656]. By analyzing these positions, we

were able to identify the following alleles: *1, *2, *3, *4, *5, *6, *9, *10, *17, *29, *34, *35, *39, and *41.

We included a pre-amplification step for genomic material using a commercial primer pool (Applied Biosystems) specific for amplifying the CYP2D6 gene to obtain the initial DNA quantity required for OpenArray. Initially, DNA from all samples was quantified using a NanoDrop spectrophotometer and then subjected to pre-amplification following the manufacturer's instructions, which recommend DNA samples with concentrations between 0.4 and 4 ng/µL. The final reaction volume was 5 µL, containing 2.5 µL of TaqMan PreAmp MasterMix (Applied Biosystems), 1.25 µL of OpenArray PreAmp Pool (Applied Biosystems), and 1.25 µL of genomic DNA. The amplification conditions were: 95°C for 10 minutes, followed by 14 cycles of 95°C for 15 seconds, 60°C for 4 minutes, and 99.9°C for 10 minutes. The reactions were performed using a Veriti Thermal Cycler (Applied Biosystems).

The polymorphisms were genotyped using custom OpenArray™ slides containing the assays for the 12 targets. All amplification reactions were performed in a total volume of 3.5 µL, including 2 µL of TaqMan™ OpenArray™ Genotyping Master Mix (Applied Biosystems) and 1.5 µL of product from the pre-amplification reaction (≈40 ng/µL). PCR conditions were pre-established by the manufacturer. Amplification and fluorescence detection were performed on the Applied Biosystems QuantStudio 12k Flex system (Applied Biosystems). The results were analyzed using the QuantStudio 12k Flex software v1.3.

The *CYP2D6* gene copy number was determined by qPCR using the Hs00010001_cn assay (Applied Biosystems) to evaluate gene deletions/duplications. All reactions were performed following the previous protocol published by Silvino et al [8]. Gene copy number was determined by comparing the amplification of the target gene (CYP2D6) to a reference gene (human RNase P) (Applied Biosystems), previously described as non-polymorphic for duplications and deletions. Prior to the experiment, the concentration of genomic DNA was adjusted using SpeedVac to ensure all samples had a concentration of 2.5 ng/µL.

All amplification reactions were performed in the presence of 0.5 µL of the Hs00010001_cn assay, 5.0 µL of TaqMan® 2x Universal PCR Master Mix (Applied Biosystems), 0.5 µL of Copy Number Assay (Applied Biosystems), 2 µL of water, and 2 µL of DNA (≈10 ng/µL). CYP2D6 copy number detection was performed in triplicate using 384-well plates (10 µL total reaction volume per well). PCR conditions were: 95°C for 10 minutes, followed by 40 cycles of 95°C for 15 seconds and 60°C for 60 seconds.

Amplification and fluorescence detection were performed using the ViiA7 Real-Time PCR System (Applied Biosystems) at the Real-Time PCR Platform of the René Rachou Institute – Fiocruz Minas (RPT09D). The

results were subsequently analyzed using CopyCaller® software v2.0. Copy number calls were generated using CopyCaller® Software v2.0 (Applied Biosystems) based on ΔCt comparisons between the target and reference genes. Although the method detects deletions and duplications, it does not allow for the resolution of hybrid alleles, which is a known limitation of qPCR-based CNV analysis for *CYP2D6*.

Translation of *CYP2D6* genotypes into predicted phenotypes according to the AS model

Haplotype inference was conducted using PHASE software v.2.1, running the algorithm with a burn-in of 50,000 iterations followed by 400,000 iterations, with a thinning interval of 1,000. To ensure convergence and reproducibility, the algorithm was run in duplicate, yielding identical haplotype assignments across both runs. The inferred haplotypes were then compared to the reference allele definitions curated by the Pharmacogene Variation Consortium (https://www.pharmvar.org/gene/CYP2D6) to assign *CYP2D6* star (*) alleles. Each allele was assigned a metabolic activity value based on its function: fully functional alleles (e.g., **1*, **2*) received a value of 1.0; decreased-function alleles (e.g., **9*, **10*, **17*, **29*, **41*) received scores of 0.25 or 0.5; and non-functional alleles (e.g., **3*, **4*, **5*, **6*) were assigned a score of 0.0. The sum of the two allelic activity scores yielded the individual's Activity Score (AS), which was used to infer the genotype-predicted metabolic phenotype. Individuals were classified as poor metabolizers (AS = 0), intermediate metabolizers (AS of 0.25 to 1.0), normal metabolizers (AS of 1.25 to 2.25), or ultrarapid metabolizers (AS > 2.25). The "g" prefix was used to denote that the phenotype was inferred based on genotypic data rather than measured phenotypically. This classification was applied consistently in all downstream analyses of CYP2D6 activity. In a few cases, we could not predict the CYP2D6 phenotype; however, it was possible to determine the activity score and infer enzyme activity. In these cases, both alleles and copy number variation were known.

Supplementary Table 1. Frequencies of CYP2D6 alleles in the study population.

CYP2D6 Alleles	CYP2D6 Activity[a]	Frequency (n)
**1*	Normal	0.3770 (984)
**2*	Normal	0.2233 (583)
**34*	Normal	0.0252 (66)
**35*	Normal	0.0229 (60)
**39*	Normal	0.0003 (1)
*17x2	Normal	0.0003 (1)
**9*	Decreased	0.0386 (101)
**10*	Decreased	0.0122 (32)
**17*	Decreased	0.0187 (49)
**29*	Decreased	0.0003 (1)
**41*	Decreased	0.0379 (99)
*9x2	Decreased	0.0007 (2)
*10x2	Decreased	0.0003 (1)
*41x2	Decreased	0.0003 (1)
**3*	None	0.0038 (10)
**4*	None	0.1122 (293)
**5*	None	0.0272 (71)
**6*	None	0.0015 (4)
*4x2	None	0.0022 (6)
*1x2	Increased	0.0114 (30)
*2x2	Increased	0.0122 (32)
*35x2	Increased	0.0003 (1)
Others[a]	ND	0.1849 (182)

Abbreviation: ND, not determined

n = number of chromosomes, n = 2610.

[a] CYP2D6 metabolic activity according to Pharmvar.

[b] Duplications that could not be unambiguously assigned to an allele in some heterozygous individuals.

Supplementary Table 2. Predicted CYP2D6 phenotype frequencies in the Mâncio Lima population.

**CYP2D6 Phenotype**	**Activity Score (AS)**	**n (%)**	**95% Confidence Interval**
gPM	0	53 (4.1)	2.9 - 5.1
gIM	0.25 - 1	307 (23.5)	21.2 - 25.8
gNM	1.25 - 2.25	796 (61.0)	58.3 - 63.6
gUM	>2.25	117 (9.0)	7.4 - 10.5
Indeterminate		32 (2.4)	1.6 - 3.2
**Activity Score Group[a]**			
AS ≤ 1		944 (72.3)	
AS >1		361 (27.7)	

[a] In some cases, the CYP2D6 phenotype could not be predicted, but it was possible to determine the activity score and infer enzyme activity. In these cases, both alleles and the copy number variation were known.

Supplementary Table 3. Prevalence of *Plasmodium vivax* infections screened by quantitative PCR (qPCR) in Mâncio Lima, Brazil, 2018-2021.

**Survey**	**Total qPCR tests**	**qPCR positivity, n (%)**	**Symptomatic[a], n (%)**	**Asymptomatic, n (%)**	**Proportion of asymptomatic (%)**
April-May 2018 (wave 1)	528	41 (7.77)	5 (0.95)	36 (6.82)	36/41 (87.8)
September-October 2018 (wave 2)	880	67 (7.61)	16 (1.82)	51 (5.80)	51/67 (76.1)
May-June 2019 (wave 3)	915	58 (6.34)	11 (1.20)	47 (5.14)	47/58 (81.0)
September-October 2019 (wave 4)	979	36 (3.68)	3 (0.31)	33 (3.37)	33/36 (91.7)
October-November 2020 (wave 5)	967	33 (3.41)	1 (0.10)	32 (3.31)	32/33 (97.0)
April-May 2021 (wave 6)	1004	17 (1.69)	1 (0.10)	16 (1.59)	16/17 (94.1)
October-November 2021 (wave 7)	1036	14 (1.35)	1 (0.10)	13 (1.25)	13/14 (92.9)

[a] Any symptoms (fever, chills or headache) within the past 7 days

Supplementary Table 4. Factors associated with *Plasmodium falciparum* infection in the Mâncio Lima cohort, from 2018 to 2021.

Variable	Odds Ratio	95% CI	*P*-value[a]
**Study wave number and dates**			
1, April-May 2018	*Reference*		
2, September-October 2018	1.630	0.765, 3.475	0.2057
3, May-June 2019	0.971	0.437, 2.160	0.9433
4, September-October 2019	0.527	0.217, 1.281	0.1573
5, October-November 2020	0.387	0.148, 1.011	0.0527
6, April-May 2021	0.237	0.079, 0.710	0.0101
7, October-November 2021	0.175	0.054, 0.573	0.0040
**Wealth index quartile**			
1, poorest	*Reference*		
2	0.443	0.233, 0.842	0.0130
3	0.378	0.184, 0.775	0.0079
4, wealthiest	0.397	0.191, 0.825	0.0134

Abbreviation: CI, confidence interval

[a] By generalized additive model with mixed effects at both individual and household levels, adjusted for wealth status and survey period. The model includes a smooth spline term for age based on CYP2D6 status. Infection is defined as a positive genus-specific PCR result confirmed by species-specific quantitative PCR probe-based assay, regardless of any symptoms. A total of 6302 from 1303 participants of 610 households were included in the model, after excluding participants with missing data.

Supplementary Table 5. *Plasmodium vivax* densities by quantitative PCR (amplicon copies/µL).

		Impaired CYP2D6		Normal CYP2D6
**Age category**	**n**	**Geometric Mean, Copies/µL (95% CI)**	**n**	**Geometric Mean, Copies/µL (95% CI)**
<15	8	37.15 (11.94, 115.58)	30	21.62 (10.53, 44.40)
15-34	19	15.05 (6.82, 33.25)	109	16.65 (11.16, 24.86)
35-54	16	9.88 (4.34, 22.48)	39	66.78 (26.53, 168.13)
>55	8	14.56 (3.63, 58.48)	16	24.09 (7.98, 72.72)
P-value[a]		0.373		0.103

Abbreviation: CI, confidence interval

[a] Parasitemia levels were compared between CYP2D6 groups using the Kruskal-Wallis test.

Supplementary Table 6. Analysis of association between CYP2D6 and parasite density in *Plasmodium vivax* infections.

Variable	Model 0 β coefficient[a]	95% CI	*P*-value	Model 1 β coefficient[b]	95% CI	*P*-value
**Study wave number and dates**						
1, Apr-May 2018	*Reference*			*Reference*		
2, Sep-Oct 2018	-0.247	-0.626, 0.132	0.2081	-0.290	-0.660, 0.081	0.1369
3, May-June 2019	-0.203	-0.596, 0.189	0.3164	-0.301	-0.690, 0.085	0.1405
4, Sep-Oct 2019	-0.371	-0.813, 0.071	0.1045	-0.381	-0.806, 0.048	0.0908
5, Oct-Nov 2020	-0.345	-0.807, 0.118,	0.1479	-0.451	-0.910, - 0.008	0.0566
6, Apr-May 2021	-0.647	-1.229, - 0.065	0.0321	-0.807	-1.374, - 0.232	0.0077
7, Oct-Nov 2021	0.169	-0.412, 0.750	0.5723	0.050	-0.509, 0.621	0.8659
**Wealth index quartile**						
1, poorest	*Reference*			*Reference*		
2	-0.106	-0.413, 0.201	0.4975	-0.098	-0.397, 0.194	0.5278
3	0.326	-0.043, 0.692	0.0833	0.396	0.028, 0.751	0.0366
4, wealthiest	0.340	-0.100, 0.778	0.1327	0.416	0.001, 0.832	0.0617

**Age group (years)**						
0-14	*Reference*			*Reference*		
15-34	-0.198	-0.572, 0.176	0.3020	-0.191	-0.586, 0.207	0.3649
35-54	0.140	-0.283, 0.565	0.5170	0.358	-0.099, 0.821	0.1415
>55	-0.139	-0.662, 0.386	0.6040	-0.152	-0.740, 0.445	0.6263
**CYP2D6 status**						
Normal	*Reference*			*Reference*		
Impaired	-0.18212	-0.498, 0.134	0.2600	0.169	-0.560, 0.902	0.6621
**Age group x CYP2D6 status**						
0-14 x Normal	...			*Reference*		
15-34 x Impaired	...			-0.134	-1.003, 0.726	0.7693
35-54 x Impaired	...			-1.009	-1.925, - 0.096	0.0383
>55 x Impaired	...			-0.451	-1.539, 0.624	0.4308

Abbreviation: CI, confidence interval

[a] Results for unadjusted analysis are presented under “model 0”. Number of observations: 245.

[b] Results for linear mixed model (LMM) with mixed effects at individual level, adjusted for age group, survey period and wealth status. The best fitted model included an interaction term between age group and CYP2D6 status. The final model was based on 245 observations. Residual plots and simulation-based tests indicated no significant deviations from model assumptions. The dispersion test showed no evidence of heteroskedasticity (P = 0.4320), and residuals were approximately uniform (P = 0.1505) with no excess of outliers (P = 0.7228).

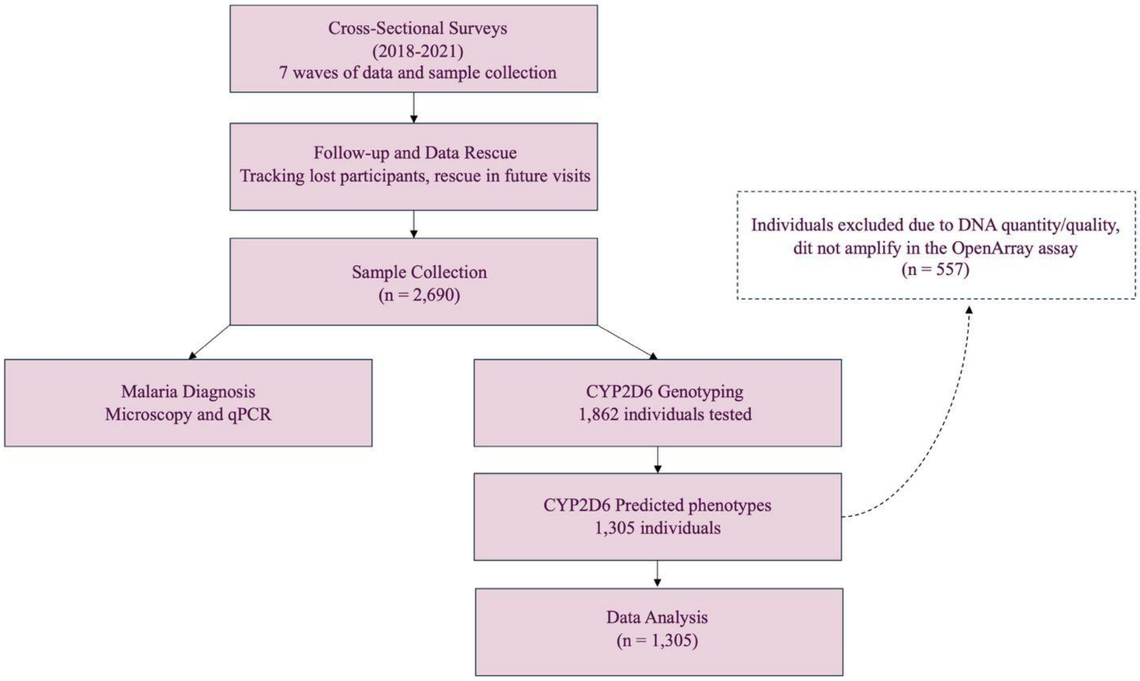

Supplementary Figure 1. Flow chart of the study participants. Overview of participant recruitment, follow-up, and inclusion in the final analysis. The chart details the number of individuals screened, excluded, and retained at each stage of this study. Inclusion criteria for cohort enrollment are described in detail in Johansen et al. (2021) [3].

REFERENCES

1. Rodrigues PT, Johansen IC, Ladeia WA, et al. Lower Microscopy Sensitivity with Decreasing Malaria Prevalence in the Urban Amazon Region, Brazil, 2018–2021. Emerg Infect Dis. **2024**; 30(9).
2. Corder RM, Paula GA, Pincelli A, Ferreira MU. Statistical modeling of surveillance data to identify correlates of urban malaria risk: A population-based study in the Amazon Basin. PLoS One. **2019**; 14(8):e0220980.
3. Johansen IC, Rodrigues PT, Tonini J, Vinetz J, Castro MC, Ferreira MU. Cohort profile: the Mâncio Lima cohort study of urban malaria in Amazonian Brazil. BMJ Open. **2021**; 11(11):e048073.
4. Corder RM, Arez AP, Ferreira MU. Individual variation in Plasmodium vivax malaria risk: Are repeatedly infected people just unlucky? PLoS Negl Trop Dis. **2023**; 17(1):e0011020.
5. Stadler E, Cromer D, Mehra S, et al. Population heterogeneity in Plasmodium vivax relapse risk. PLoS Negl Trop Dis. **2022**; 16(12):e0010990.
6. Dal'Asta AP, Lana RM, Amaral S, Codeço CT, Monteiro AMV. The Urban Gradient in Malaria-Endemic Municipalities in Acre: Revisiting the Role of Locality. Int J Environ Res Public Health. **2018**; 15(6):1254.
7. Silva-Nunes M da, Ferreira MU. Clinical spectrum of uncomplicated malaria in semi-immune Amazonians: beyond the " symptomatic " vs " asymptomatic " dichotomy. Mem Inst Oswaldo Cruz. **2007**; 102(3):341–348.
8. Silvino ACR, Kano FS, Costa MA, et al. Novel Insights into Plasmodium vivax Therapeutic Failure: CYP2D6 Activity and Time of Exposure to Malaria Modulate the Risk of Recurrence. Antimicrob Agents Chemother. **2020**; 64(5).
9. Ladeia-Andrade S, Menezes MJ, Sousa TN de, et al. Monitoring the Efficacy of Chloroquine-Primaquine Therapy for Uncomplicated Plasmodium vivax Malaria in the Main Transmission Hot Spot of Brazil. Antimicrob Agents Chemother. **2019**; 63(5).
10. Gaedigk A, Ingelman-Sundberg M, Miller NA, Leeder JS, Whirl-Carrillo M, Klein TE. The Pharmacogene Variation (PharmVar) Consortium: Incorporation of the Human Cytochrome P450 CYP Allele Nomenclature Database. Clin Pharmacol Ther. **2018**; 103(3):399–401.
